# Attitude of young psychiatrists toward coercive measures in psychiatry: a case vignette study in Japan

**DOI:** 10.1186/1752-4458-3-20

**Published:** 2009-09-22

**Authors:** Masaru Tateno, Kanna Sugiura, Kumi Uehara, Daisuke Fujisawa, Yueren Zhao, Naoki Hashimoto, Hidehiko Takahashi, Naofumi Yoshida, Takahiro Kato, Wakako Nakano, Yosuke Wake, Tomohiro Shirasaka, Seiju Kobayashi, Soichiro Sato

**Affiliations:** 1 Department of Neuropsychiatry, School of Medicine, Sapporo Medical University, South-1, West-16, Chuo-ku, Sapporo, Japan 0608543; 2 Department of Psychiatry, Yokohama City University, Yokohama, Japan; 3 Department of Psychiatry, Kanagawa Psychiatric Center Serigaya Hospital, Yokohama, Japan; 4 Department of Psychiatry, School of Medicine, Keio University, Tokyo, Japan; 5 Department of Psychiatry, Meisei Hospital, Kumamoto, Japan; 6 Department of Psychiatry, Hokkaido University Graduate School of Medicine, Sapporo, Japan; 7 Department of Molecular Neuroimaging, Clinical Neuroimaging Section, National Institute of Radiological Sciences, Chiba, Japan; 8 Department of Neuropsychiatry, Toho University, School of Medicine, Tokyo, Japan; 9 Department of Neuropsychiatry, Graduate school of Medical Sciences, Kyushu University, Fukuoka, Japan; 10 Department of Psychiatry, University of Occupational and Environmental Health, Kitakyushu, Japan; 11 Department of Neuropsychiatry, Okayama University Graduate School of Medicine, Dentistry and Pharmaceutical Sciences, Okayama, Japan; 12 Department of Psychiatry, Zikei Hospital, Okayama, Japan

## Abstract

**Background:**

Every psychiatrist must pay careful attention to avoid violating human rights when initiating coercive treatments such as seclusion and restraint. However, these interventions are indispensable in clinical psychiatry, and they are often used as strategies to treat agitated patients. In this study, we investigated young psychiatrists' attitudes toward psychiatric coercive measures.

**Methods:**

A total of 183 young psychiatrists participated as subjects in our study. A questionnaire with a case vignette describing a patient with acute psychosis was sent to the study subjects via the Internet or by mail. This questionnaire included scoring the necessity for hospitalization, and the likelihood of prescribing seclusion and/or restraint, on a 9-point Likert scale (with 9 indicating strong agreement).

**Results:**

There was general agreement among the study subjects that the case should be admitted to a hospital (8.91 ± 0.3) and secluded (8.43 ± 1.0). The estimated length of hospitalization was 13.53 ± 6.4 weeks. Regarding the likelihood of prescribing restraint, results showed great diversity (5.14 ± 2.5 on 9-point scale); psychiatrists working at general hospitals scored significantly higher (6.25 ± 2.5) than those working at university hospitals (5.02 ± 2.3) or psychiatric hospitals (4.15 ± 2.6). A two-group comparison of the length of inpatient care revealed a significant difference between those psychiatrists who scored 1-3 (n = 55, 14.22 ± 7.4 wks) and those who scored 7-9 (n = 62, 12.22 ± 4.0) regarding the need to use restraint.

**Conclusion:**

Our results may reflect the current dilemma in Japanese psychiatry wherein psychiatrists must initiate coercive measures to shorten hospitalization stays. This study prompted its subject psychiatrists to consider coercive psychiatric treatments.

## Background

There have always been concerns about human rights infringements for coercive psychiatric measures, such as involuntary admission, forced medication, seclusion and/or restraint [1]. Controlled studies have provided no evidence about the validity of such interventions, primarily because ethical considerations make it difficult to perform randomized controlled trials [2,3]. However, such involuntary treatments are indispensable in many clinical practice scenarios, and they are commonly used as strategies to treat patients exhibiting disruptive and violent behaviors [3-7].

The Mental Health Act in Japan was initially passed on May 1, 1950, and was originally called the Mental Hygiene Law. In 1988, the Mental Hygiene Law was revised and renamed the Mental Health Law. In 1995, the current version, the Mental Health and Welfare Law, came into force [8-10]. All psychiatrists practicing in Japan must abide by this law, which provides for the fundamental human rights of people with psychiatric problems.

The Mental Health and Welfare Law defines three types of admission: voluntary hospitalization, hospitalization for medical care and protection, and involuntary hospitalization ordered by a prefectural governor [11]. In Japan, the judicial process does not become involved in decision-making about involuntary hospitalizations. Instead, the Japanese government empowers designated physicians for mental health to be entrusted with safeguarding the rights of subjects with psychiatric conditions. Designated physicians also have the right and duty to initiate and terminate coercive measures such as seclusion and restraint.

In 1998, after the disclosure of human rights violations in some Japanese psychiatric hospitals and in response to pressing social demand, Asai et al. conducted a national survey about involuntary psychiatric treatments and published a detailed report and guidelines the following year [12]. Asai and his collaborators distributed survey sheets to 1,548 hospitals with psychiatric beds and received 1,090 responses (70.4 percent), suggesting an increasing interest in this topic. After this survey and elaborate analysis, official guidelines on restraint and seclusion were published by the Japanese Society of General Hospital Psychiatry's educational committee [13]. The issuance of these guidelines deepened clinical psychiatrists' awareness of behavioral restrictions and educated practitioners about the importance of these measures as potential therapeutic strategies in psychiatric emergencies. However, opportunities for studying psychiatric seclusion and restraint are limited when compared to opportunities to study pharmacotherapy or psychotherapy.

The aim of this survey was to learn Japanese psychiatrists' attitudes about emergency interventions for acute psychosis by focusing on involuntary treatments and exploring the possibility of minimizing psychiatric coercive measures.

## Methods

### Subjects

The subjects of this study were 183 young Japanese psychiatrists. Site investigators were recruited through the Japan Young Psychiatrists Organization's (JYPO; ) listserv, and those site investigators in turn encouraged their colleagues to participate in the survey. We provided three options for answering the questionnaire: online, email, or conventional mail. The study authors mailed a questionnaire to site investigators at the collaborating institutes. The site investigators physically distributed the questionnaire to their colleagues or sent an email with the URL and login password for the online questionnaire. All subjects were requested to complete the questionnaire during the survey period, January 1 to February 28, 2009. The purpose of this study was clearly stated on the cover sheet of the questionnaire and answering the questionnaire was considered to be consent. All responders participated in this study without any incentive. Similarly, all authors and subjects involved in this study declared themselves free of any conflict of interest relating to the study.

### Questionnaire Contents

The questionnaire consisted of a case vignette and questions in three categories: (1) the use of hospitalization; (2) the length of inpatient care, and (3) the use of seclusion and/or restraint [see Additional file 1]. After reading the case vignette, all respondents were asked to score the need for involuntary hospitalization, identify the type of admission, estimate the length of inpatient care, and the likelihood of prescribing seclusion and/or restraint.

The questionnaires were returned anonymously. However, respondents were asked to provide demographic information regarding their levels of psychiatric experience, the types of facilities in which they worked, the region in which they practice, and whether they were designated mental health physicians. The questionnaire is shown in the Appendix.

### Statistical Analysis

Study results were expressed as mean ± SD. Statistical analysis was performed using SPSS 16.0J for Windows (SPSS Japan Inc., Tokyo, Japan). A student's t-test and ANOVA were applied, respectively, for the comparisons of two groups and three or more groups. The statistical significance was set at a p value of less than 0.05.

## Results

A total of 183 young psychiatrists answered this study's questionnaire. We collected data from all seven regions in Japan, with relatively higher rates in Hokkaido/Tohoku and Kyushu. Because we used three different methods of data collection (online, email, or conventional mail), it was difficult to calculate a precise total response rate. The response rate for the email attachments and conventional mail was 93.3% (n = 112). However, several factors complicated the response rate calculation for the Internet data collection because some mailing lists used in this study contained a number of invalid addresses. Based on the estimated response rate reported by each site investigator, we estimated the total response rate at approximately 85%. Because of a defect in the questionnaire sheet as distributed during the earliest stage of this study, for 65 out of 183 respondents (35.5 percent) it was impossible to connect scores on a 9-point scale and the psychiatrists' length of clinical experience. The average length of psychiatric experience was 7.49 ± 5.6 (mean ± SD) years (n = 118). The rate of designated physicians was 50.8 percent. Designated mental health physicians had significantly greater clinical experience (11.30 ± 5.2 years, n = 60) as compared to non-designated psychiatrists (3.45 ± 2.2, n = 58). For the type of facility, 103 of the survey participants worked at university hospitals, 36 at general hospitals, and 34 at psychiatric hospitals, and the remaining 10 respondents worked at psychiatric clinics, academic schools, or public health facilities.

The study results were summarized in tables. Almost all respondents (98.9 percent) scored 7 or higher regarding the need for hospitalization, including 162 psychiatrists who scored 9 (88.5 percent) as shown in Table 1. Most respondents scored 7 or higher on a 9-point Likert scale regarding the likelihood of prescribing seclusion (8.43 ± 1.0), whereas the scores regarding prescribing restraint displayed a greater diversity (5.14 ± 2.5).

**Table 1:** The need for hospitalization, its form and length, and the likelihood of prescribing seclusion and/or restraint.

**Necessity of hospitalization**	**9 point scale (9 = strongly agree)**	
Overall (n = 183)	8.91 ± 0.3	
Designated physician for mental health (n = 60)	8.85 ± 0.5	p = 0.28
Non-designated physician (n = 58)	8.93 ± 0.3	

**Form of admission**	Out of 183 respondents	
Voluntary Hospitalization	0	
Medical Care and Protection	77 (42.1%)	
Ordered by Prefectural Governor	104 (56.8%)	
No answer	2 (1.1%)	

**Estimated length of hospitalization**	Weeks	
Overall (n = 183)	13.53 ± 6.4	
Designated physician for mental health (n = 60)	14.07 ± 7.3	p = 0.31
Non-designated physician (n = 58)	12.88 ± 5.0	

**Likelihood of seclusion**	9 point scale (9 = strongly agree)	
Overall (n = 182)	8.43 ± 1.0	
Designated physician for mental health (n = 59)	8.51 ± 0.9	p = 0.35
Non-designated physician (n = 58)	8.33 ± 1.2	

**Likelihood of restraint**	9 point scale (9 = strongly agree)	
Overall (n = 183)	5.14 ± 2.5	
Designated physician for mental health (n = 60)	4.98 ± 2.5	p = 0.37
Non-designated physician (n = 58)	5.40 ± 2.4	

Survey results are expressed with a mean ± SD. P values were calculated with a Student's t-test between the two subgroups. No statistically significant differences were found.

Regarding the likelihood of prescribing restraint, a two-group comparison between designated and non-designated physicians demonstrated no significant difference. However, psychiatrists working at general hospitals did score significantly higher (6.25 ± 2.5) than those who work at university hospitals (5.02 ± 2.3) or psychiatric hospitals (4.15 ± 2.6) as illustrated in Table 2.

**Table 2:** Comparing the likelihood of prescribing restraint and the estimated hospitalization length.

**Likelihood of Restraint**	**9 point scale** **(9 = strongly agree)**	
Overall (n = 183)	5.14 ± 2.5	

Designated mental health physician (n = 60)	4.98 ± 2.5	p = 0.37^1^
Non-designated physician (n = 58)	5.40 ± 2.4	
University hospital (n = 103)	5.02 ± 2.3	
General hospital (n = 36)	6.25 ± 2.5	p = 0.03^2^
Psychiatric hospital (n = 34)	4.15 ± 2.6	p = 0.001^3^

**Estimated length of hospitalization**	weeks	
Agreed with restraint (score 7-9, n = 62)	12.22 ± 4.0	p = 0.049
Disagreed with restraint (score 1-3, n = 55)	14.22 ± 7.4	

Survey results are expressed with a mean ± SD. p values were calculated using Student's t-test between the two subgroups. Significant differences were found between those psychiatrists practicing in general hospitals and the two other types of hospitals. No significant variation was found between psychiatrists in university and psychiatric hospitals.

Significance of difference between:

^1 ^Designated mental health physician and Non-designated physician

^2 ^University hospital and general hospital

^3 ^General hospital and psychiatric hospital

We divided the survey respondents into two groups, based on scores regarding the likelihood of prescribing restraint: those psychiatrists who favored restraint (score 7-9) and those who were opposed (score 1-3). Those psychiatrists who favored the use of restraint were found to estimate significantly shorter periods of inpatient care (12.22 ± 4.0) than those professionals who opposed restraint (14.22 ± 7.4).

## Discussion

Every psychiatrist must pay careful attention to avoid violating human rights when initiating coercive treatments such as seclusion and restraint. However, these interventions are indispensable in clinical psychiatry, and they are often used as strategies in the treatment of agitated patients.

The Mental Hygiene Law was intended to protect the fundamental human rights of people with mental illness and facilitate their rehabilitation within the community. Since enactment of the law in 1950, all psychiatric medical professionals in Japan have been bound to practice psychiatry with careful consideration to avoid infringing upon human rights. There have been certain calls from a humanitarian viewpoint for the abolition of seclusion and restraint. However, in acute psychiatry, these coercive measures can be useful therapeutic strategies to ensure the safety of psychiatric patients [3-7]. In Japan, judgment regarding the necessity for involuntary psychiatric admission is entrusted to designated mental health physicians. The judicial system never becomes involved in this decision-making process. In order to admit a patient for hospitalization to provide medical care and protection, a designated physician obtains written consent from that patient's guardian [11,14].

Article 29 of the Mental Health and Welfare Law states that if a prefectural governor recognizes that a person who has been examined is diagnosed as mentally disordered and is therefore likely to hurt himself/herself or others unless hospitalized for medical care and protection, the prefectural governor may admit the person to a mental hospital established by the national or prefectural government or a designated hospital. This form of forced hospitalization can be approved only when the person has been examined by at least two designated physicians and the examination results of each physician conclude that the person is mentally disordered and that he or she is likely to hurt himself/herself or others because of a mental disorder unless admitted to a hospital for medical care and protection.

In Japan, there is no uniform residency program in each medical specialty. Instead of standardized training programs, there is a two-tier psychiatric training system in Japan: (1) specialist certification by the Japanese Society of Psychiatry and Neurology; and (2) government designation. To become a designated mental health physician, applicants for designation must have clinical experience exceeding five years, including over three years in general psychiatry. Designated mental health physician candidates must take a three-day course of lectures and submit eight case reports of involuntary hospitalization in six categories: schizophrenia (three case reports including at least one case in which the patient was admitted by a prefectural gubernatorial order, which is the most coercive type of hospitalization), mood disorder, substance abuse, dementia, organic disorders, and child and adolescent mental health. Thus, the main purpose of this designation system is to thoroughly acquaint psychiatrists with the Mental Health Law and authorize psychiatrists to execute various involuntary interventions based on Japan's strict mental health regulations.

According to the results of the present study, the average score ranking the necessity of hospitalization was 8.91 ± 0.3 on the 9-point Likert scale, with 98.9 percent of respondents scoring a 7 or higher. With regard to the form of admission, opinions were nearly divided in half: 42.1 percent responded that hospitalization for medical care and protection would be most likely, whereas 56.8 percent said an involuntary hospitalization ordered by a prefectural governor would be a likely type of admission. In the case vignette used in this study, Mr. A. brandished a kitchen knife and threatened his neighbors. This behavior may be considered to satisfy the legal requirements for involuntary hospitalization. However, in real life situations, hospitalization for medical care and protection, a less coercive measure, is more commonly suggested. The polarization of the respondents' opinions on this point might be attributable to differences in their interpretations of the case vignette.

There was significant diversity among the respondents' estimations of hospitalization length, which ranged from four weeks (n = 4) to one year (n = 1). The majority of respondents suggested twelve weeks (n = 106), with an average of 13.53 ± 6.4 weeks. Two group comparisons between the designated mental health physicians and the non-designated physicians revealed no statistically significant difference between the two groups' estimations of hospitalization length. Further, no correlations were found between the estimated hospitalization length and the likelihood of prescribing restraint, nor were correlations discovered between the estimated hospitalization length and the length of physicians' psychiatric experience. However, the two group comparisons between psychiatrists who favored restraint and those who opposed it revealed that those practitioners who favored restraint suggested a significantly shorter hospitalization length than those who opposed restraint. We cannot provide a clear explanation for this result. The result might indicate that restraint is considered an outcome of treatments that target earlier improvement in the manifestation of psychiatric symptoms. Hoge et al. reported that most episodes of refusal to take antipsychotic medication by consumers ended with voluntary acceptance of treatment [15]. However, it takes time to persuade patients to take oral medication and often requires additional staff. To ensure minimum coerciveness in psychiatric practice, we need additional studies to explore those factors affecting psychiatrists' decisions about initiating coercive measures.

Psychiatrists in other countries may consider a three-month hospitalization to be somewhat excessively long. However, it is noteworthy that Japan has been criticized for its lengthy hospitalization periods for schizophrenic patients [11]. When considering this national mental health care backdrop, the three-month hospitalization suggested in this study certainly reflects the recent improvements in Japanese psychiatrists' awareness about shortening hospital stay durations. In the treatment case presented, the patient lives alone and has no prior history of psychotic episodes. Unfortunately, Japan still suffers from a lack of social resources enabling people with mental disorders to live within their communities. Further measures are needed to shorten the length of hospital stays.

For employing seclusion versus restraint, the score for the likelihood of prescribing seclusion showed a high concurrence rate among the respondents, with an average of 8.43 ± 1.0 on a 9-point scale. Alternatively, the score for the likelihood of prescribing restraint ranged from 1 to 9, with an average of 5.14 ± 2.5. In Japan, seclusion in a room with a certain amount of space and equipped with a bathroom is considered less restrictive than restraint. At a previously held international workshop on seclusion and restraint that we organized, we realized through discussions with psychiatrists from other countries that cultural backgrounds would influence psychiatrists' opinions about behavioral restrictions [16]. For instance, when the Czech Republic became a target of criticism because of their use of a cage bed--a bed surrounded by a metal cage used to restrain a patient--the Czechs explained that in the Czech Republic the use of a "net bed" was considered more humane than other restraint techniques, such as straps, isolation rooms, or even strong medication. It is important to understand that differences in psychiatric opinions may be due to differences between cultural backgrounds [17].

When comparing scores for estimated hospitalization lengths, according to the types of hospitals where physicians work, those who work at general hospitals suggested a significantly longer period than those who work at university hospitals or psychiatric hospitals. One reason for this result could be explained by the psychiatric departments in most general hospitals being understaffed while having a higher percentage of patients requiring restraint, for example, people who are sent to the emergency room with an altered level of consciousness or delirium patients with comorbid physical conditions. Another reason could be that there are increasing numbers of patients with behavioral and psychological symptoms of dementia (BPSD) resulting from the rapid aging of the Japanese population. Yet another reason for expecting a longer hospitalization period at general hospitals might be the nurses' working environment. It has been reported that training nurses is effective in decreasing the number of behavioral restrictions at hospitals [18,19]. However, certain nursing system characteristics in the psychiatric wards of many general hospitals could be hindering this effect. For instance, nurses in general hospitals are routinely transferred to different wards after a certain period of time and therefore are likely to be less experienced, tending to resign sooner because of their workload.

As for limitations of this survey, the questionnaire was sent to the subjects with a brief description of an imaginary case rather than a real patient. The subjects of this study represent only a subset of psychiatrist in Japan. The latest data provided by the Japanese Ministry of Health, Labor and Welfare reports that the total number of psychiatrists was 12,474, accounting for 4.49% of all medical doctors in 2006 (on-line database of JMHLW; ). The number of doctors under the age of 40 was 93,409 in 2006. Considering these data, we estimated the number of young psychiatrists as 4,194. Thus, the subjects of this study account for 4.36% of all young Japanese psychiatrists. Similarly, the number of designated physicians for mental health was 11,791 in 2006. Our sample included only 0.5% of those designated physicians, indicating limited representation. In regard to the 9-point scale used in this study, a 5 score indicates neither agreement nor disagreement on a 9-point Likert scale (with 9 being the highest possible score) and the significance of the deviation from the mean of 5 remains controversial. Therefore, it is difficult for us to draw firm conclusions.

## Conclusion

In recent years, many studies have been conducted on psychiatric seclusion and restraint, especially in Europe [20-22]. It has been reported that some programs have succeeded in reducing restraint [23,24]. A previous study revealed that experiencing coercion during admission negatively affected patients' attitudes toward treatment and adherence to medication [25]. We believe that psychiatrists early in their careers should consider how to minimize the use of behavioral restrictions. It is feasible that early training determines the subsequent clinical custom of each psychiatrist. Going forth into clinical duties with this in mind will no doubt shorten the hours of seclusion and restraint for current and future patients.

## Competing interests

The authors declare that they have no competing interests.

## Authors' contributions

The members of Coercive Treatment in Psychiatry Study Group of Japan Young Psychiatrists Organization (MT, KS, KU, DF, YZ, NH, HT, NY, SS) designed the study protocol and collected data in collaboration with site investigators (TK, WN, YW, TS, SK). All authors had full access to the data. MT performed the statistical analysis and drafted the manuscript. All authors have read and approved the final manuscript.

## Supplementary Material

Additional file 1 **Questionnaire**. The questionnaire with case vignette used in this study. Click here for file
