# Supplementary material for: Attitude of young psychiatrists toward coercive measures in psychiatry: a case vignette study in Japan
Source: Int J Ment Health Syst. 2009 Sep 22;3:20. doi: 10.1186/1752-4458-3-20 (PMC2754431; doi:10.1186/1752-4458-3-20)
Supplement: Additional file 1 — Questionnaire. The questionnaire with case vignette used in this study. [file 1752-4458-3-20-S1.DOC]

**Attitude of young psychiatrists toward coercive measures in psychiatry: A case vignette study in Japan**

Masaru Tateno^1^,**^§^**, Kanna Sugiura^2^, Kumi Uehara^3^, Daisuke Fujisawa^4^, Yueren Zhao^5^, Naoki Hashimoto^6^, Hidehiko Takahashi^7^, Naofumi Yoshida^8^, Takahiro Kato^9^, Wakako Nakano^10^, Yosuke Wake^11^, Tomohiro Shirasaka^1^, Seiju Kobayashi^1^, Soichiro Sato^12^

# Questionnaire

| **Case vignette**  Mr. A, a Japanese male, is in his thirties and is of average height and build. He has no previous history of psychiatric conditions. Mr. A gradually isolated himself and then refused to be contacted by his neighbors. He began to claim that “they are going to attack me with a radio wave from outer space”. He stayed at home alone most of the time after sealing up all the windows. One day, Mr. A entered his neighbor’s house and suddenly started to scream “You are the one! You are one of the aliens!” His talk was disorganized and he held a kitchen knife pointing at his neighbor. He showed distinct delusion and agitation, and had no sense of being mentally ill. Mr. A was brought to your hospital.  You saw Mr. A in an examination room and diagnosed that he was suffering from acute psychosis with delusions and agitation. In the exam room, he yelled at you saying “You must be one of them!” and was about to hit you. He has no insight and keeps refusing your interventions persistently. Your hospital has psychiatric inpatient beds including currently unoccupied seclusion rooms. What will be your typical way of dealing with this case as a psychiatrist? |
| --- |

Please answer on a 9-point Likert scale ranging from 1=strongly disagree to 9=strongly agree, and 5=neither agree nor disagree.

Q1. He refuses admission for inpatient care because he lacks insight.

Does he need to be admitted to psychiatry ward against his will?

What is your answer on a 9-point scale (9=strongly agree)? [ ]

Q2. What will be the most likely type of admission? [ ]

(1) Voluntary Hospitalization, (2) Hospitalization for Medical Care and Protection,

(3) Involuntary Hospitalization Ordered by Prefectural Governor,

(4) Others (Be specific, ), (5) I cannot answer

Q3. What will be the estimated length of hospitalization?

[ day(s)/week(s)/month(s) ]

Q4. Should the patient be secluded?

In this context, seclusion means the involuntary placement of an individual alone in a locked room simply equipped with a bed and a toilet.

What is your answer on a 9-point scale (9=strongly agree)? [ ]

Q5. Should the patient be performed restraint?

In this context, restraint is the fixation of at least one of the patient’s limbs by a mechanical appliance (mechanical restraint).

What is your answer on a 9-point scale (9=strongly agree)? [ ]

Please describe yourself.

How long is your experience in psychiatry (as a psychiatrist) [ years]

[ ] click here if you are a designated physician for mental health.

At what type of hospital do you work? [ ]

(1) University hospital, (2) General hospital, (3) Psychiatry hospital, (4) Psychiatry clinic, (5) Others (Be specific, )

In which region do you practice? [ ]

(1) Hokkaido/Tohoku, (2) Kanto, (3) Chubu, (4) Kinki, (5) Chugoku/Shikoku,

(6) Kyushu/Okinawa
